# A scanning probe microscopy study of nanostructured TiO_2_/poly(3-hexylthiophene) hybrid heterojunctions for photovoltaic applications

**DOI:** 10.3762/bjnano.9.197

**Published:** 2018-08-01

**Authors:** Laurie Letertre, Roland Roche, Olivier Douhéret, Hailu G Kassa, Denis Mariolle, Nicolas Chevalier, Łukasz Borowik, Philippe Dumas, Benjamin Grévin, Roberto Lazzaroni, Philippe Leclère

**Affiliations:** 1 Laboratory for Chemistry of Novel Materials - Center for Innovation and Research in Materials and Polymers - CIRMAP, University of Mons, Mons, Belgium; 2 Aix Marseille Univ, CNRS, Centre Interdisciplinaire de Nanoscience de Marseille (CiNaM), Marseille, France; 3 Materia-Nova R&D Center, Mons, Belgium; 4 Universitè Grenoble Alpes, F-38000 Grenoble, France; 5 CEA, LETI, Campus MINATEC, F-38054 Grenoble, France; 6 UMR5819 SYMMES CEA-CNRS-UGA, 17 rue des Martyrs F-38054, Grenoble, France

**Keywords:** hybrid heterojunctions, hybrid photovoltaic, Kelvin probe force microscopy, photoconductive-AFM, photo-KPFM, poly(3-hexylthiophene), TiO_2_

## Abstract

The nanoscale morphology of photoactive hybrid heterojunctions plays a key role in the performances of hybrid solar cells. In this work, the heterojunctions consist of a nanocolumnar TiO_2_ surface covalently grafted with a monolayer of poly(3-hexylthiophene) (P3HT) functionalized with carboxylic groups (–COOH). Through a joint analysis of the photovoltaic properties at the nanoscale by photoconductive-AFM (PC-AFM) and surface photovoltage imaging, we investigated the physical mechanisms taking place locally during the photovoltaic process and the correlation to the nanoscale morphology. A down-shift of the vacuum level of the TiO_2_ surface upon grafting was measured by Kelvin probe force microscopy (KPFM), evidencing the formation of a dipole at the TiO_2_/P3HT-COOH interface. Upon in situ illumination, a positive photovoltage was observed as a result of the accumulation of photogenerated holes in the P3HT layer. A positive photocurrent was recorded in PC-AFM measurements, whose spatial mapping was interpreted consistently with the corresponding KPFM analysis, offering a correlated analysis of interest from both a theoretical and material design perspective.

## Introduction

Over the past decades, a large range of photovoltaic (PV) technologies have been developed for the production of renewable energy [1]. Inorganic photovoltaic cells are currently the most employed PV devices with a power efficiency ranging from 20 to 40% [2] and a long-term stability up to 20 years [3]. However, a number of drawbacks affect those technologies. Indeed, in addition to high energy consumption for their fabrication, these devices are deposited on rigid substrates and involve relatively heavy and costly materials of possibly low abundance and/or toxicity [4]. New PV technologies, such as organic photovoltaics (OPV) and hybrid solar cells, are now being developed [2] to cope with such issues. In particular, hybrid solar cells can possibly benefit from the low economic and energy costs of production, high absorbance and tailorable absorption spectrum of the organic materials on the one hand, and from the good stability, absorption and electrical properties of the inorganic materials on the other hand.

Hybrid PV devices include various technologies such as perovskite cells, dye sensitized solar cells (DSSC), with power efficiencies up to 13% [5] and hybrid bulk heterojunctions (HBHJ), which combine an organic matrix and inorganic semiconducting nanostructures such as quantum dots. Among the electron acceptor materials commonly used for DSSC and HBHJ, titanium dioxide (TiO_2_) is a well-known metal oxide semiconductor [6–8]. Depending on its nanostructure and its crystalline phase, its conductivity varies from 10^−4^ Ω^−1^·cm^−1^ to 10^−11^ Ω^−1^·cm^−1^ [9–10]. TiO_2_ is very valuable because it can easily form nanostructures, such as nanoporous layers, nanowires or nanocolumns [5,11–12]. Because of its large band gap (3.2 eV [13]), light absorption is carried out by an organic or inorganic dye. The nanostructuration of the acceptor material is crucial for the cell performance [11], as it allows increasing the specific surface of the layer to enhance the amount of grafted dye, and thereby, the photon absorption yield. Nanostructuration is also likely to improve the conductivity of TiO_2_ [14]. Because of the influence of the nanostructuration of TiO_2_ on the optoelectronic properties of the device, it is of prime interest to study the photovoltaic properties at the nanoscale. Hybrid heterojunction (HHJ) structures are obtained by impregnation of the porous layer with an absorbing dye or a polymer electron donor. Poly(3-hexylthiophene) (P3HT) is often used, because of its strong absorption, its high hole mobility and its donor-like electronic properties [15]. Upon light absorption by the polymer, excitons are generated and they can be dissociated at the interface with TiO_2_, the polymer also acting as the hole-transporting layer.

In this work, we investigated nanostructured TiO_2_ layers composed of arrays of nanoscale columns, covalently sensitized with a P3HT-COOH monolayer to form hybrid bulk heterojunctions. The grafting of P3HT on the surface of TiO_2_, ensured by the COOH groups, was demonstrated to be beneficial for the photoconversion efficiency of the system [16–18]. The vertically aligned nanostructuration of TiO_2_ also makes this system attractive, since it ensures direct percolation paths for the photogenerated electrons from the donor–acceptor interface to the cathode, while providing a simple, controlled and ordered architecture. Furthermore, studies are available in literature regarding the photovoltaic response of TiO_2_/P3HT blends [16–23] and can be used as a reference for meaningful interpretations of our measurements, both in terms of photocurrent and photovoltage under illumination. The columnar TiO_2_/P3HT-COOH HHJs have been studied by photoconductive-AFM (PC-AFM) and photo-assisted Kelvin probe force microscopy (photo-KPFM) to follow the photovoltaic response, i.e., photocurrent and photovoltage, respectively, at the nanoscale under illumination, in order to understand the local physical processes taking place during the photoconversion of energy, and their correlation with the nanoscale morphology of the active layer. A key aspect of this work consists in the joint analysis of these correlated PC-AFM and KPFM measurements, providing a more fundamental understanding of the photovoltaic mechanisms at stake in the systems. To the best of our knowledge, this joint KPFM/PC-AFM study of such a nanostructured array of TiO_2_ columns sensitized with functionalized P3HT-COOH constitutes a novel result of interest from both a theoretical and material design perspectives.

## Materials and Methods

**Figure 1:**
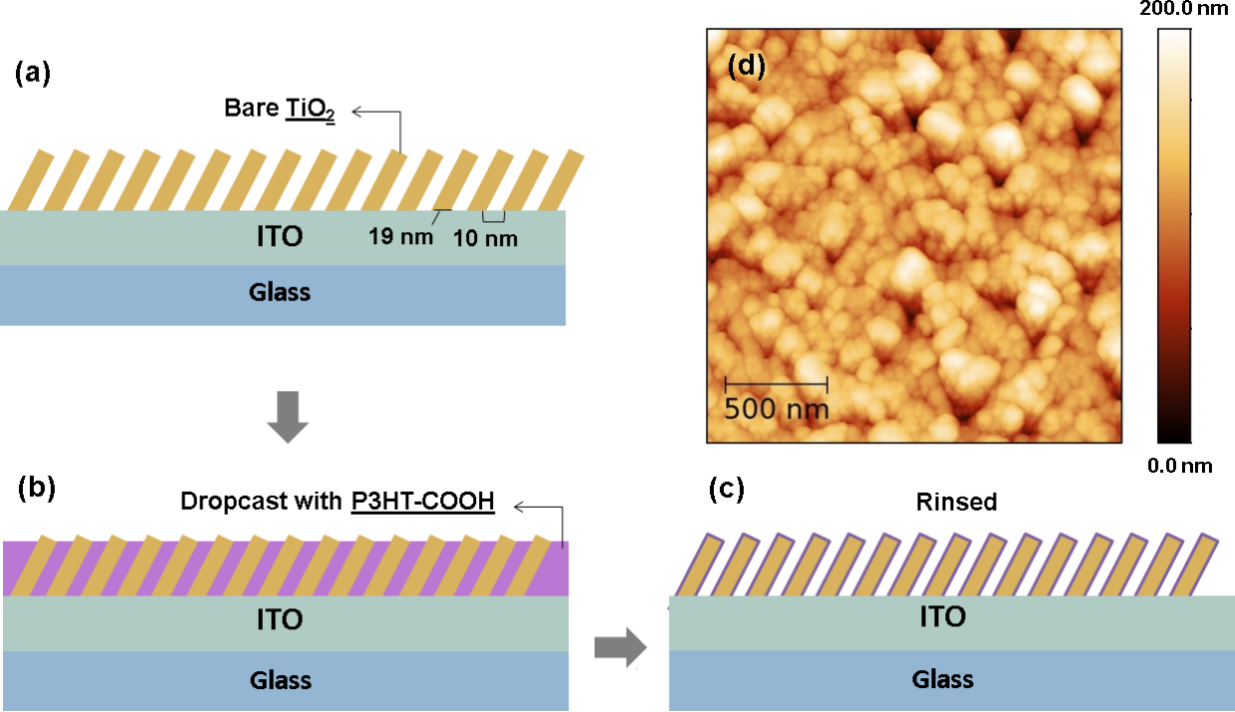
(a–c) Schematic representation of the TiO_2_/P3HT-COOH HHJ preparation. (d) 2 × 2 µm^2^ TM-AFM height image of a bare nanocolumnar TiO_2_ layer.

The TiO_2_ layers were synthesized by magnetron sputtering in grazing mode. A thorough description of the fabrication process can be found in the literature [24], which also identified the optimized fabrication parameters for prospective photovoltaic applications. In compliance with these recommendations, the layers were synthesized without any substrate rotation or bias, while fixing the growth temperature to 450 °C and the tilt angle between the substrate and the cathode axis to 60°. Anatase TiO_2_ layers with a 200 nm thick nanocolumnar morphology have been deposited on 85 nm-thick ITO-coated glass substrates (Naranjo B.V., sheet resistance of 15 Ω·sq). The average spacing between the columns is (10 ± 3) nm, with an average width of the columns of (19 ± 4) nm, as determined by SEM measurements [24]. The topography of the deposit is shown in the tapping-mode atomic force microscopy (TM-AFM) image of Figure 1d, where the apex of the columns appears as hemispherical protuberances. Regio-regular P3HT-COOH (5400 g/mol, which corresponds to about 30 monomer units, i.e., a total polymer chain length around 130 Å) was synthesized following a reported procedure [24]. A schematic description of the grafting protocol is given in Figure 1a–c. The polymer deposit was obtained by dropcasting a 0.5 mg/mL solution of P3HT-COOH in chlorobenzene on the TiO_2_ structure. The covalent grafting of the polymer on the nanoporous TiO_2_ surface is ensured by the carboxylic –COOH group. Rinsing with chlorobenzene was then carried out to remove the residual ungrafted polymer chains. The success of the polymer grafting is confirmed by UV–visible optical absorption measurement across a 350–800 nm wavelength range, for which an absorption of light higher by one order of magnitude compared to bare TiO_2_ was measured [24]. This indicates a good P3HT impregnation along the columns, the interspacing being sufficient for the polymer infiltration.

The photo-KPFM measurements were carried out in a UHV (<10^−10^ Torr) instrument composed of an Omicron Nanotechnology VT-AFM system with a Nanonis controller. The KPFM electrical excitation used a frequency ω_KPFM_/2π of 958 Hz, with a VAC amplitude of 600 mV. The light source for sample irradiation was a green laser diode (wavelength = 500 nm, power density = 1.45 mW/mm^2^). Photo-assisted KPFM measurements were also performed in ambient conditions, with a Bruker multimode microscope controlled by a Nanoscope III unit coupled to a Nanonis control unit (SPECS Zürich). The KPFM electrical excitation was made at a frequency ω_KPFM_/2π of 80 Hz, with a VAC amplitude of 500 mV. The illumination of the sample for photo-KPFM and photovoltage probing was provided by a white light lamp irradiating the sample surface from the top. In both setups, conductive Nanosensors PPP-EFM tips (PtIr-coated Si probes) were used (resonant frequency around 75 kHz). The sample was grounded while the excitation and regulation biases were applied to the tip. The measured contact potential difference (*V*_cpd_) is given by the following expression:

[1]
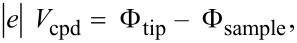


where Φ_tip_ and Φ_sample_ are the workfunction of the tip and the sample, respectively. In this work, no calibration of the tip workfunction was necessary, as only the *V*_cpd_ variations between the materials constituting the photovoltaic blends and their modifications with incoming light were to be measured. These *V*_cpd_ variations provide relative but quantitative variations of surface potential at the investigated interfaces.

The PC-AFM measurements were carried out in air, using a Bruker Dimension Icon microscope with a Nanoscope V controller. An extended TUNA external module was used for current detection with a detection range within 100 fA to 1 µA. Silicon tips coated with a PtIr conductive alloy (PPP-CONTPt from Nanosensors) were used. The tip and the back-contact were connected while the sample was locally irradiated from the bottom (through the patterned ITO–glass substrates) under AM 1.5 calibrated white light illumination (spot diameter around 200 µm, power density of 100 suns).

## Results and Discussion

### Photo-KPFM measurements on the TiO_2_/P3HT-COOH hybrid heterojunctions

#### Analysis of the *V*_cpd_ contrast in the dark

**Figure 2:**
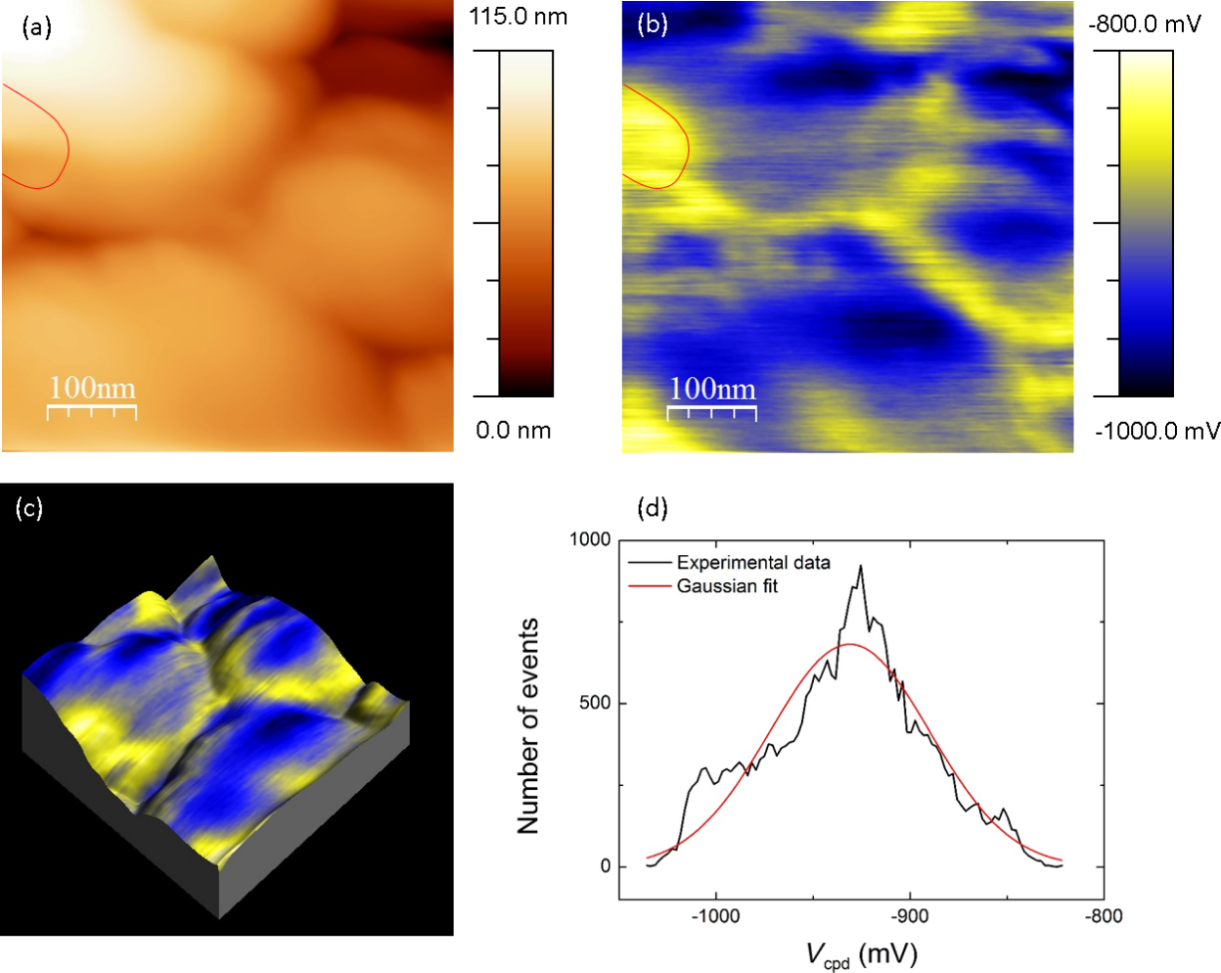
(a) 500 × 500 nm^2^ AFM height image of a nanostructured TiO_2_ film, obtained in UHV. Height detection parameters: −22 Hz of frequency shift setpoint and 40 mV of amplitude setpoint. (b) Corresponding KPFM *V*_cpd_ image. *V*_cpd_ detection parameters: frequency and amplitude of the electrical excitation: 958 Hz and 600 mV. (c) Three-dimensional display of the height image (a), shown with the color scale of the *V*_cpd_ (b). (d) *V*_cpd_ histogram extracted from (b) (black line) superimposed with a Gaussian fit (red curve).

Figure 2a shows a 500 × 500 nm^2^ AFM height image obtained in UHV on a nanocolumnar TiO_2_ film deposited over a grounded ITO electrode, where the nanocolumns of TiO_2_ are assembled in clumps with a width of several hundred nm. Figure 2b shows the corresponding KPFM *V*_cpd_ image. Figure 2c presents the three-dimensional display of Figure 2a, where the colour scale refers to the *V*_cpd_ signal of Figure 2b. The distribution of the *V*_cpd_ values can be fit with a Gaussian distribution centred at −931 mV with a FWHM of 97 mV (Figure 2d).

**Figure 3:**
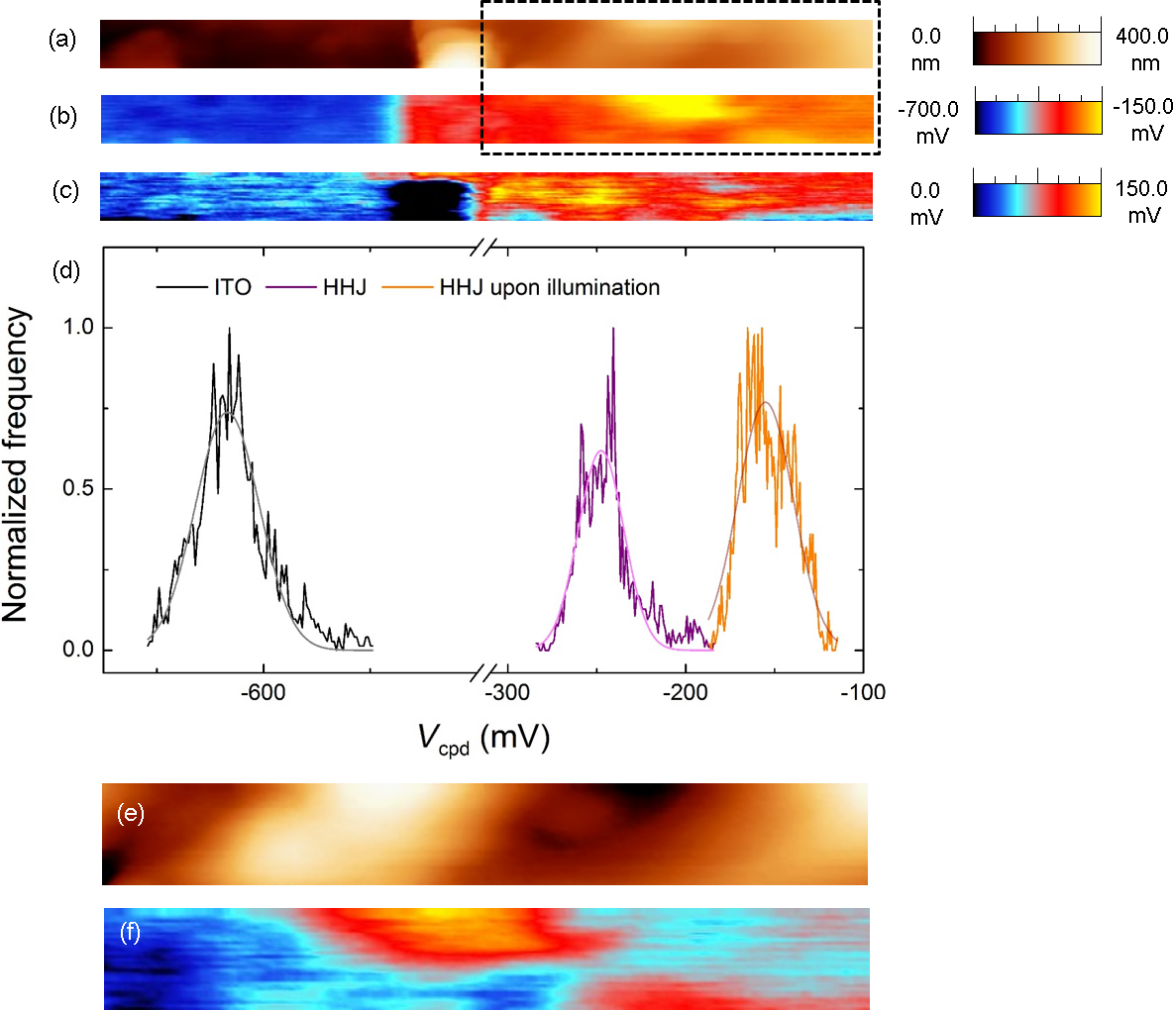
(a) AFM height image obtained in UHV over a 4000 × 270 nm^2^ scan area astride the step edge between a TiO_2_/P3HT-COOH HHJ (right-part of the image) and the uncovered part of the ITO electrode (left-part of the image). KPFM height detection parameters: −5 Hz of frequency shift setpoint and 50 mV of amplitude setpoint. (b) Corresponding KPFM *V*_cpd_ image recorded without illumination. KPFM *V*_cpd_ detection parameters: frequency and amplitude of the electrical excitation: 958 Hz and 600 mV. (c) Corresponding KPFM *V*_ph_ image obtained by subtracting the *V*_cpd_ images recorded with and without illumination. (d) *V*_cpd_ distributions across a 250 × 500 nm^2^ surface area in the left (ITO area) and right (HHJ area) part of image (a) and in the right (HHJ area) part of image (c). Gaussian fits have been added for each distribution. (e) and (f) are enlargements of images (a) and (b), respectively, corresponding to the dashed rectangle. The colour scale contrast is enhanced to highlight the main features.

A direct correlation between the topography and the *V*_cpd_ signal can be observed, with a higher height corresponding to a more negative *V*_cpd_. It is however unlikely that the contrast purely originates from a crosstalk between the topography and the *V*_cpd_, as indicated by local mismatching between both contrasts (see red lines in Figure 2a and 2b). Moreover, further measurements (see Figure 3) showed that P3HT grafting barely affects the overall morphology but smooths tremendously the *V*_cpd_ contrast. Thus, the observed *V*_cpd_ contrast most probably originates therefore from local variations in the electronic properties of the surface, such as a possibly different free electron density at the top and at the side of the columns. This explanation is further supported by the PC-AFM measurements presented in the last section. As shown in the Supporting Information File 1 (Figure S1), no ungrounded potential is to be detected at the top of the nanocolumnar TiO_2_ film. This can therefore not be the origin of the *V*_cpd_ contrast observed on the bare TiO_2_ columns.

Figure 3a displays a KPFM height image obtained in UHV on a TiO_2_ deposit grafted with P3HT-COOH. The left part of the image corresponds to a bare area of the ITO electrode, while the right part shows a TiO_2_/P3HT-COOH zone. Figure 3b shows the corresponding *V*_cpd_ image recorded in the dark. A clear difference between the *V*_cpd_ intensity over the ITO electrode (−614 ± 18 mV in average) and the TiO_2_/P3HT-COOH HHJ (−248 ± 49 mV in average) is observed. This *V*_cpd_ shift clearly appears in the *V*_cpd_ distributions of Figure 3d. The more negative *V*_cpd_ value over the ITO electrode reflects consistently a higher corresponding work function (around 4.7 eV in literature [6,15]) compared to that of TiO_2_ (around 4.3 eV in literature [7]).

The data of Figure 3 were compared with the images obtained on bare nanocolumnar TiO_2_ (Figure 2). In both measurements, the ITO electrode was grounded and the same tip was used. The distribution of *V*_cpd_ values on the TiO_2_/P3HT-COOH area (right part of Figure 3b) is displayed as the purple curve in Figure 3d; the corresponding Gaussian fit is centred at −248 mV, with a FWHM of 30 mV. As seen in Figure 2d, the *V*_cpd_ is much more negative on bare nanocolumnar TiO_2_, This indicates that: (i) the P3HT layer induces an up-shift of the *V*_cpd_ values, and (ii) this up-shift occurs over the entire surface, since no values typical of bare TiO_2_ are recorded on the polymer-grafted surface. This indicates that the P3HT covering is complete, with no bare TiO_2_ area left. The fact that the *V*_cpd_ increases upon P3HT grafting indicates that the surface workfunction of TiO_2_/P3HT-COOH is lower than that of bare nanocolumnar TiO_2_. This can be understood on the basis of the following discussion, which describes the relative configuration of the electronic levels of the materials within the HHJ.

The covalent bonding between P3HT-COOH and TiO_2_ creates a dipole at the interface induced by: (i) the hybridization of the electronic orbitals of the two components, leading to a rearrangement of the charge density at the interface and (ii) the addition of a net dipole intrinsic to the P3HT-COOH molecule itself. The first effect was reported previously [25], evidencing a pinning of the LUMO of P3HT-COOH at the conduction band of the TiO_2_ with a net transfer of half an electron per polymer chain from the LUMO of P3HT into the CB of TiO_2_. This results in the formation of a dipole at the P3HT-COOH/TiO_2_ interface, directed away from TiO_2_, where the positive (negative) pole is located in P3HT (TiO_2_). Previous KPFM studies [26–28] confirmed the presence of a dipole directed away from TiO_2_ or ITO substrates upon grafting of COOH-containing organic materials. A dipole directed away from the TiO_2_ surface (i.e., a negative dipolar moment) means a downshift of the vacuum level upon grafting [29].

The local variations in the *V*_cpd_ values (the FWHM of the distribution is about 30 mV) are probably due to slightly different densities of grafted P3HT-COOH chains. Indeed, a homogeneous P3HT covering would induce a homogeneous up-shift of the *V*_cpd_ across the surface, leading to a variation range of *V*_cpd_ for the TiO_2_/P3HT-COOH HHJ having the same origin as that of bare nanocolumnar TiO_2_. Yet, unlike what was observed for bare nanocolumnar TiO_2_, no correlation between the height and *V*_cpd_ images can be seen between Figure 3e and Figure 3f. The origin of the contrast is therefore not to be linked to the *V*_cpd_ variations in the TiO_2_ surface, but rather to an inhomogeneous contribution of the grafted P3HT-COOH.

**Figure 4:**
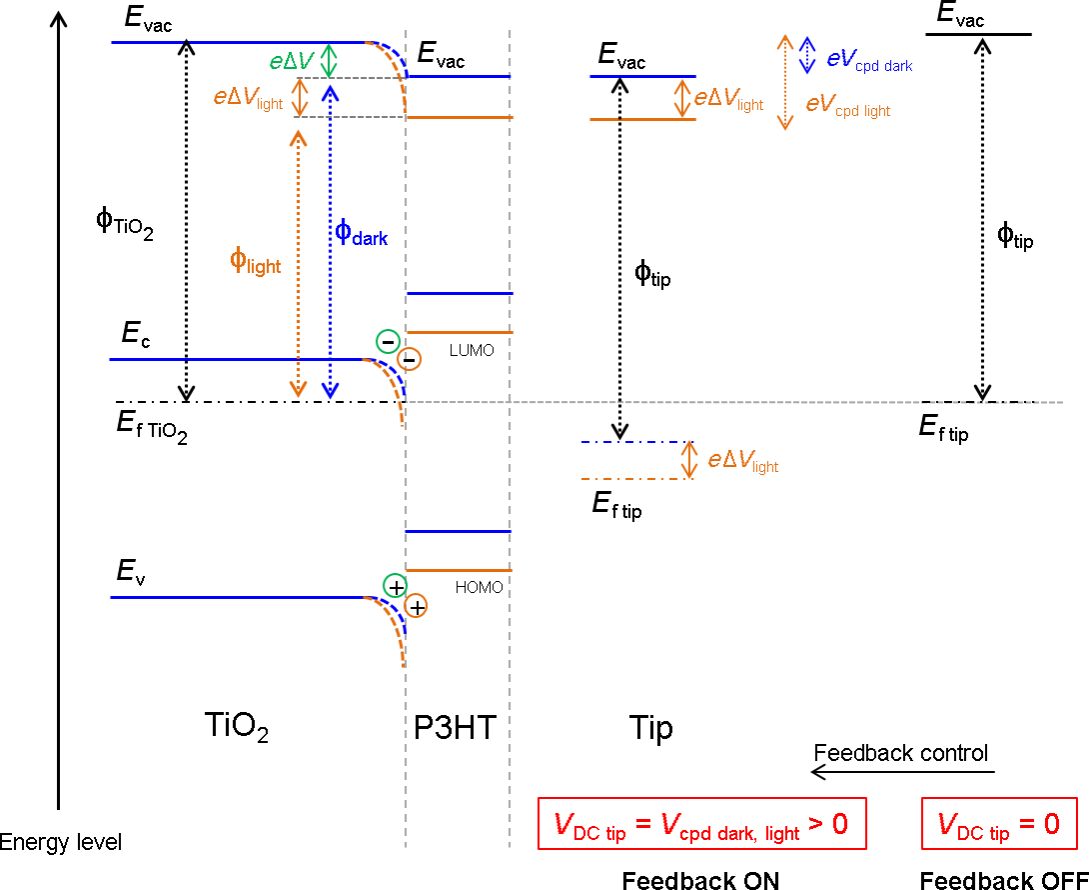
Schematic representation of the electronic band structure of the [TiO_2_/P3HT-COOH]–[tip] system_,_ in the KPFM measurement configuration considering no feedback control (right part) and feedback control (left part). The blue (orange) lines correspond to the situation in the dark (under illumination). *e*Δ*V* (green color) represents the bond dipole in the dark, while *e*Δ*V*_light_ (orange color) represents the photovoltage under illumination. *E*_vac_, *E*_c_, *E*_v_, *E*_f_ and Φ stand for vacuum level, conduction band, valence band, Fermi level and workfunction, respectively. HOMO and LUMO mean highest occupied molecular orbital and lowest unoccupied molecular orbital, respectively.

Figure 4 shows a schematic representation of the band diagram of the ITO/TiO_2_/P3HT-COOH/tip electronic system (blue lines) in a KPFM measurement configuration, i.e., a grounded ITO electrode and the DC and AC bias applied to the tip. Considering no floating potential at the [ITO/TiO_2_/P3HT-COOH] surface (see Supporting Information File 1, Figure S1), a Fermi level alignment can be assumed across the entire ITO/HHJ structure. The dipole pointing away from the TiO_2_ at the TiO_2_/P3HT-COOH interface, leading to a partial accumulation of *e*^−^ (*h*^+^) in the TiO_2_ (P3HT), will bend the vacuum level downwards, hence lowering the surface workfunction of the TiO_2_ once grafted with P3HT-COOH. The more positive *V*_cpd_ of TiO_2_/P3HT-COOH compared to bare TiO_2_ confirms this mechanism.

**Figure 5:**
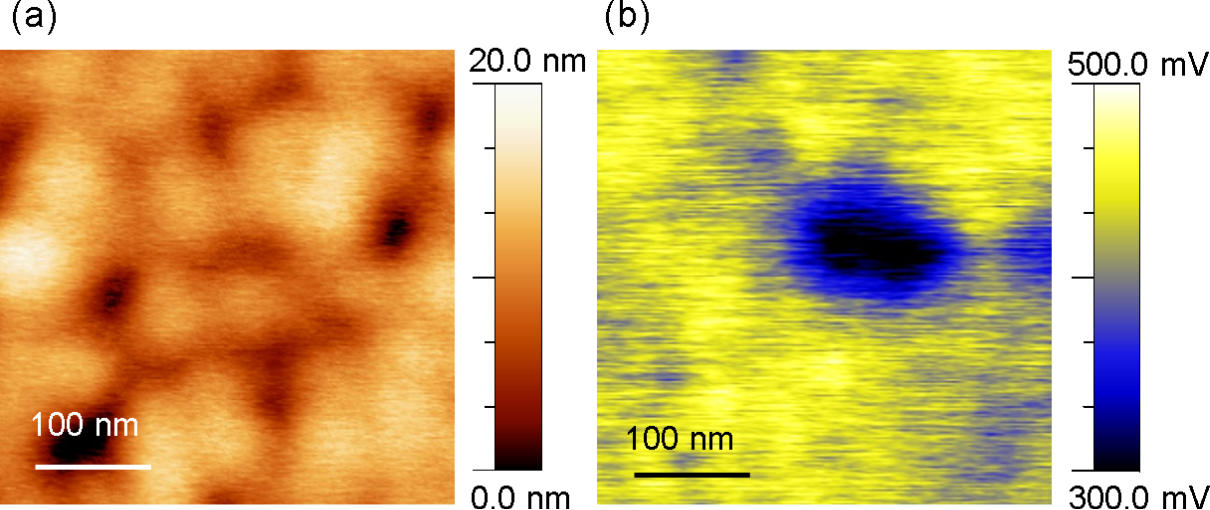
(a) 400 × 400 nm^2^ AFM height image obtained in air on a TiO_2_/P3HT-COOH HHJ. KPFM height detection parameters: −10 Hz of frequency shift setpoint and 5 nm of amplitude setpoint. (b) Corresponding KPFM *V*_cpd_ image. KPFM *V*_cpd_ detection parameters: frequency and amplitude of the electrical excitation: 80 Hz and 500 mV, respectively.

Figure 5a shows a KPFM height image obtained on a TiO_2_/P3HT-COOH deposit in ambient conditions in the dark. While the top of the TiO_2_ columns is visible, topographical features cannot be assigned to the presence of P3HT, probably because the nominal thickness of the P3HT-COOH deposit (13 nm) is similar to the roughness of the columnar assembly. No correlation is observed between the columnar topography and the corresponding surface potential image (Figure 5b), which shows variations within [260; 500] mV. By comparison with the data of Figure 2, this confirms that the *V*_cpd_ contrast is ruled by the presence of P3HT-COOH at the surface of TiO_2_. The *V*_cpd_ contrast in Figure 5b can be explained on the basis of the bond dipole at the TiO_2_/P3HT-COOH interface discussed above. *V*_cpd_ can then be expressed as *V*_cpd_ = *V*_cpd TiO2_ + *e*Δ*V*, *V*_cpd TiO2_ and *e*Δ*V* being the *V*_cpd_ of bare TiO_2_ and the local bond dipole amplitude, respectively. The lower (higher) *V*_cpd_ observed in the darker (brighter) zones in Figure 5 (b) corresponds therefore to a lower (higher) *e*Δ*V*, which could be related to a lower (higher) P3HT-COOH grafting density.

#### Variations of *V*_cpd_ upon illumination

As a preliminary study, KPFM measurements on bare TiO_2_ were carried out in the dark and upon illumination (white light). The results are presented in Supporting Information File 1, Figure S2. As expected, no photovoltage is observed, TiO_2_ being transparent in the visible spectrum.

Figure 3c shows the KPFM positive photovoltage across the entire TiO_2_/P3HT-COOH surface (right-part of the image) upon illumination. This up-shift of *V*_cpd_ upon illumination is better visualized in the corresponding profiles in Figure 3d. This photovoltage confirms locally a complete P3HT covering over the TiO_2_ surface. The positive photovoltage means an increase of the *V*_cpd_ value, i.e., a decrease of the surface workfunction. This effect can be understood on the basis of Figure 4. Upon grafting, it was previously discussed that a dipole is created at the TiO_2_/P3HT-COOH interface, with positive (negative) charges in the P3HT (TiO_2_) layer. This leads to a *V*_cpd_ value denoted *V*_cpd dark_ in Figure 4 and expressed as:

[2]
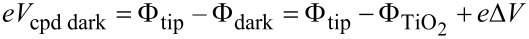


where Φ_tip_, Φ_TiO2_ and Φ_dark_ are the workfunctions of the tip, the TiO_2_ layer and the sample surface, respectively. ΔV represents the further voltage compensation needed to cancel the electrostatic forces between the tip and the sample, due to the excess positive charges present in the P3HT layer, i.e., the bond dipole. Upon illumination, it is expected that P3HT-COOH absorbs the incident photons, thus creating excitons. The length of the P3HT-COOH chains being sufficiently small, irrespective of the location where the excitons are generated, they will be able to reach the TiO_2_/P3HT-COOH interface, and dissociate by transferring an electron from P3HT into the conduction band of TiO_2_. An accumulation of holes in the highest occupied molecular orbital (HOMO) of P3HT and electrons in the conduction band of TiO_2_ follows, with the charges remaining close to the interface due to electrostatic attraction. A steady state is then reached between the generation and recombination of charges. The photogeneration of positive charges in the P3HT layer induces an additional *V*_DC_ that has to be compensated in the KPFM measurement to nullify the tip–sample electrostatic forces. This compensation is denoted Δ*V*_light_ in Figure 4, and the *V*_cpd_ value upon illumination, *V*_cpd light_, is now expressed as:

[3]
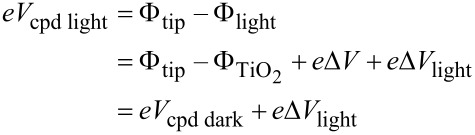


This provides the following expression for the photovoltage:

[4]
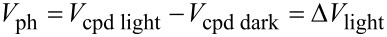


Δ*V*_light_ is a positive quantity because the DC bias applied to the tip (*V*_DC tip_) (to compensate for positive charges in P3HT) is necessarily positive. The relation between the surface potential and *V*_DC tip_ is given by *V*_cpd_ = *V*_DC tip_. This leads to a positive value of the photovoltage, as observed experimentally in Figure 3.

#### Photoconductive-AFM measurements on the TiO_2_/P3HT-COOH hybrid heterojunctions

**Figure 6:**
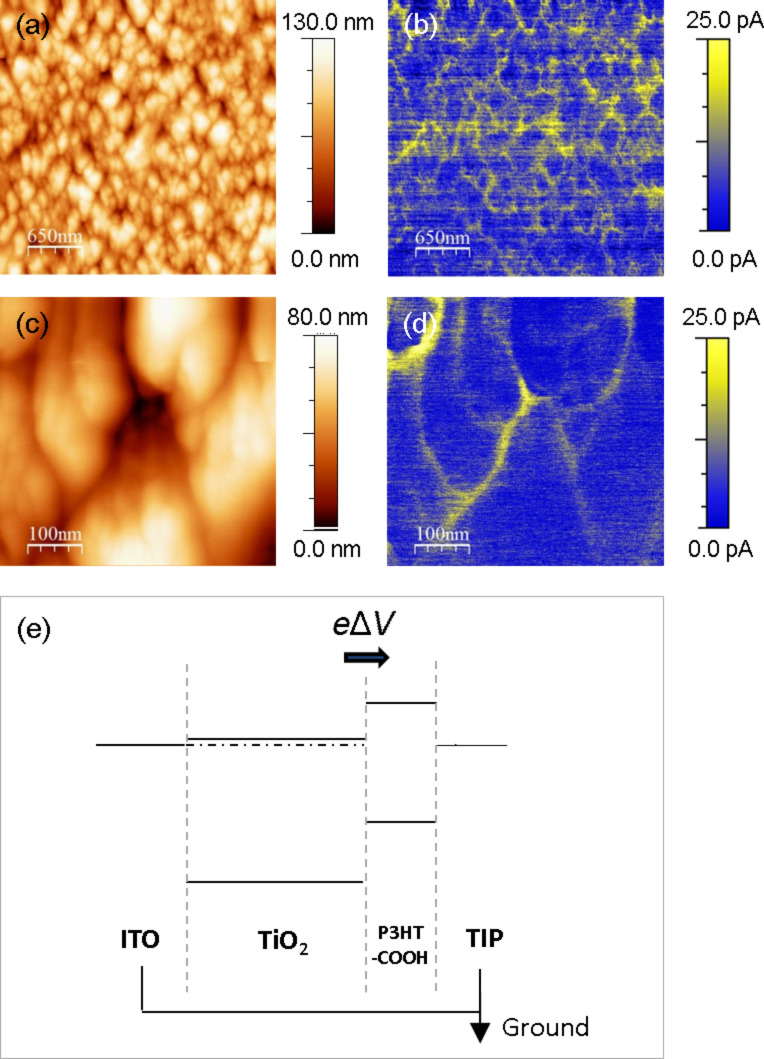
5 × 5 µm^2^ (a,b) and 500 × 500 nm^2^ (c,d) PC-AFM height and photocurrent images of a TiO_2_/P3HT-COOH HHJ. The images were recorded upon calibrated illumination (AM 1.5, 100 suns), in short-circuit configuration. (e) Schematic representation of the electronic band structure of the ITO/TiO_2_/P3HT-COOH/tip system in short-circuit configuration. *e*Δ*V* corresponds to the bond dipole.

A 5 × 5 µm^2^ height image of a TiO_2_/P3HT-COOH HHJ is shown in Figure 6a. The corresponding current image in Figure 6b, obtained in short-circuit configuration upon illumination, shows values of photocurrent up to 25 pA. This confirms light absorption by the P3HT-COOH, followed by the generation of charges at the TiO_2_/P3HT-COOH interface. The positive sign of the photocurrent means that the charges collected at the tip are holes. The generation and collection of charges upon illumination can be explained on the basis of Figure 6e, which displays the electronic band structure of the ITO/TiO_2_/P3HT-COOH/tip system in short-circuit configuration.

Upon illumination, the photon absorption by P3HT-COOH leads to the creation of excitons in the polymer. The electrons are transferred in the conduction band of TiO_2_ at the TiO_2_/P3HT-COOH interface. As the COOH group contributes to the LUMO of P3HT-COOH, the transfer of the electron to the conduction band of TiO_2_ is favored compared to unsubstituted P3HT [25]. The photogenerated holes (electrons) are collected at the tip (ITO), and a positive photocurrent is measured when probing the P3HT-COOH layer.

However, the photocurrent map of Figure 6b is far from uniform, with a positive photocurrent reaching 25 pA on the regions corresponding to the inter-columnar spaces, while it is 14 pA over the top of the columns. These local variations are highlighted in Figures 6c and 6d.

The origin of those local variations could be due to the difference in tip–sample contact area between the top of the columns and the intercolumnar zones. However, it is observed in Figure 6a and 6b that, while the topographic variations are of similar amplitude across the entire surface, the intensity of photocurrent in the areas between columns varies, and is therefore not impacted solely by the topographic variations.

We note that the *I*_ph_ contrast is qualitatively similar to that of the *V*_cpd_ observed in Figure 2, in which the top of the bare TiO_2_ nanocolumns displays more negative *V*_cpd_ values. *I*_ph_ and *V*_cpd_ quantify two different physical mechanisms, being the amount of photogenerated charges flowing in the system for the former, and the sample surface workfunction relatively to that of the tip for the latter. However, both quantities are influenced by the electron density in the conduction band of the TiO_2_ and the grafting density of P3HT-COOH. These two properties impact the local conductive properties at the tip–sample contact, thus the resulting photocurrent. Φ_TiO2_ and the P3HT-COOH grafting density are also expected to impact the resulting *V*_cpd_ since we previously expressed the latter as:


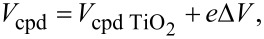


where the first and second terms are directly related to Φ_TiO2_ and the P3HT-COOH density, respectively.

Due to the small thickness of the P3HT-COOH layer on top of the TiO_2_ columns, the photocurrent contrast recorded with the tip in direct contact with the surface is most probably ruled by the TiO_2_ electrical properties. This explains why the *I*_ph_ contrast shows similarities with the *V*_cpd_ contrast of bare TiO_2_, rather than with that of the TiO_2_/P3HT-COOH HHJ. In such a configuration, the similarity of contrast between the *I*_ph_ (Figure 6b,d and *V*_cpd_ (Figure 2b,c) images suggests that the lower photocurrent measured on top of the columns might originate from a locally lower initial (i.e., prior to illumination) electron density at the TiO_2_ surface. Among various possible factors, this variation of electron density might be due to the presence of different TiO_2_ crystal facets, as the latter are shown to influence the electronic properties of the TiO_2_ surface [30–31].

## Conclusion

Nanocolumnar TiO_2_ layers were sensitized with a layer of P3HT-COOH. KPFM surface potential measurements indicate complete covering of the TiO_2_ surface by the polymer. A down-shift of the vacuum level of the sample upon grafting, i.e., an increase of the surface potential, was measured, due to the formation of a bond dipole at the TiO_2_/P3HT-COOH interface. Upon in situ illumination, a positive photovoltage was observed, which is related to the accumulation of photogenerated holes in the P3HT layer. Along with the surface potential shift, a positive photocurrent was measured by PC-AFM measurements over the TiO_2_/P3HT-COOH heterojunction upon illumination, corresponding to a hole collection at the tip. Lower photocurrent values measured on top of the TiO_2_ columns can be related to the corresponding more negative *V*_cpd_, indicating a locally lower electron density pre-existing the illumination.

## Supporting Information

File 1 Supporting Information.
Figure S1 shows a FM-AFM height image obtained in UHV astride the step from a nanostructured TiO_2_/P3HT-COOH HHJ to the ITO electrode lying below. The applied DC sample bias was varied during the measurement, without illumination. This result aims at demonstrating the absence of floating potential across the layer composing the sample. Figure S2 shows the superimposition of FM-KPFM height and *V*_cpd_ profiles over a nanostructured TiO_2_ film obtained in UHV and recorded with and without illumination. The result aimed at demonstrating the absence of light-induced artefact during the recording of topography, as well as the negligibility of the photovoltaic effect at the TiO_2_/ITO interface.
