# Supplementary material for: A scanning probe microscopy study of nanostructured TiO2/poly(3-hexylthiophene) hybrid heterojunctions for photovoltaic applications
Source: Beilstein J Nanotechnol. 2018 Aug 1;9:2087–96. doi: 10.3762/bjnano.9.197 (PMC6122299; doi:10.3762/bjnano.9.197)
Supplement: File 1 — Supporting Information. Figure S1 shows a FM-AFM height image obtained in UHV astride the step from a nanostructured TiO2/P3HT-COOH HHJ to the ITO electrode lying below. The applied DC sample bias was varied during the measurement, without illumination. This result aims at demonstrating the absence of floating potential across the layer composing the sample. Figure S2 shows the superimposition of FM-KPFM height and Vcpd profiles over a nanostructured TiO2 film obtained in UHV and recorded with and without illumination. The result aimed at demonstrating the absence of light-induced artefact during the recording of topography, as well as the negligibility of the photovoltaic effect at the TiO2/ITO interface. [file Beilstein_J_Nanotechnol-09-2087-s001.pdf]

Supporting Information

for

A scanning probe microscopy study of nanostructured TiO_2/poly(3-hexylthiophene) hybrid heterojunctions for photovoltaic applications

Laurie Letertre[1], Roland Roche[2], Olivier Douhéret[3], Hailu G. Kassa[1], Denis Mariolle[4,5], Nicolas Chevalier[4,5], Łukasz Borowik[4,5], Philippe Dumas[2], Benjamin Grévin[4,6], Roberto Lazzaroni[1,3] and Philippe Leclère[*1]

Address: [1]Laboratory for Chemistry of Novel Materials - Center for Innovation and Research in Materials and Polymers - CIRMAP, University of Mons, Mons, Belgium, [2]Aix Marseille Univ, CNRS, Centre Interdisciplinaire de Nanoscience de Marseille (CiNaM), Marseille, France, [3]Materia-Nova R&D Center, Mons, Belgium, [4]Université Grenoble Alpes, F-38000 Grenoble, France, [5]CEA, LETI, Campus MINATEC, F-38054 Grenoble, France and [6]UMR5819 SYMMES CEA-CNRS-UGA, 17 rue des Martyrs F-38054, Grenoble, France

Email: Philippe Leclère - Philippe.LECLERE@umons.ac.be

* Corresponding author

Supporting Information

Figure S1a shows a FM-AFM height image obtained in UHV over a 3800 × 400 nm² area astride the step edge between a 200 nm thick nano-structured TiO_2/P3HT-COOH HHJ (right part of the image) and the area of the ITO-glass substrate left uncovered (left part of the image).

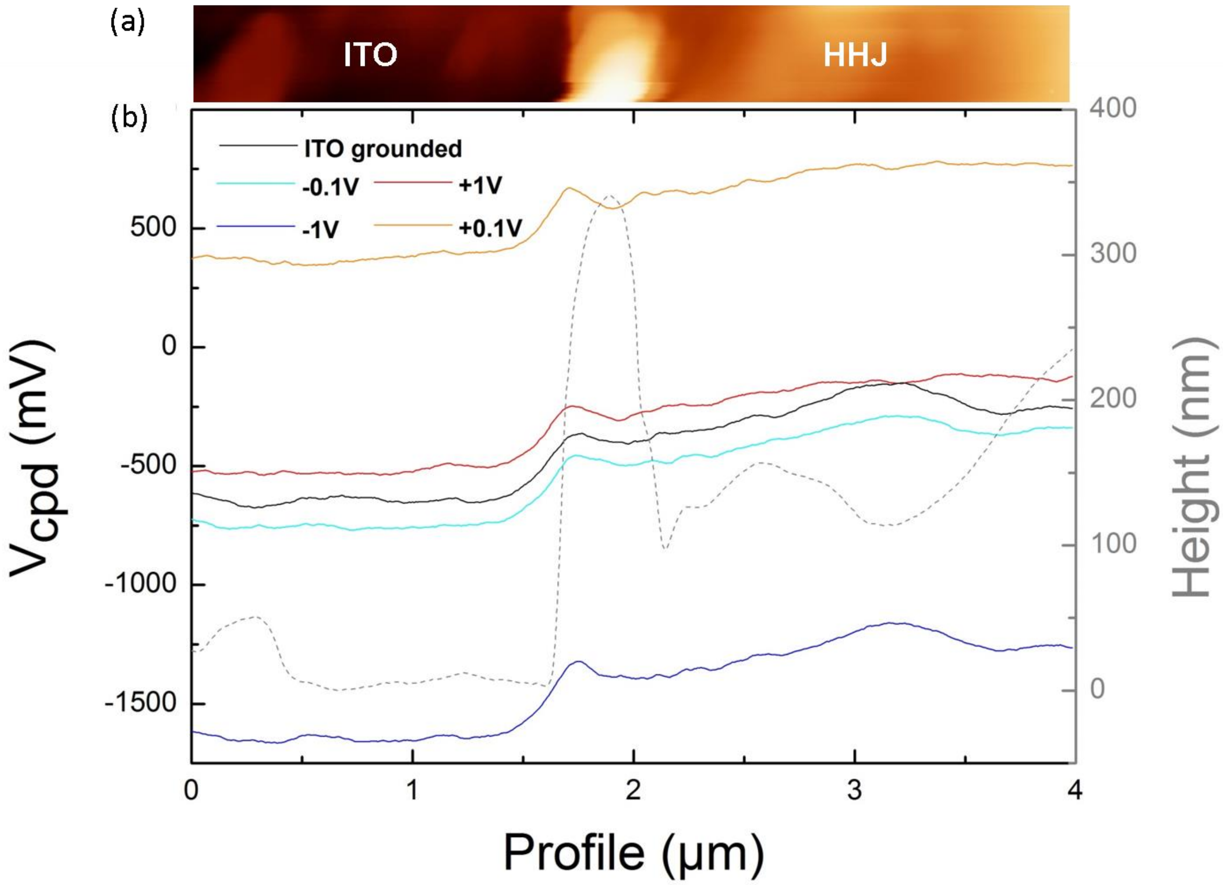

Figure S1: (a) FM-AFM height image in UHV over a 3800 × 400 nm² scan size astride the interface of a 200 nm-thick nano-structured TiO_2/P3HT-COOH HHJ (right part of the image) and the ITO electrode lying below (left part of the image). FM-AFM parameters: -5 Hz of frequency shift setpoint and 50 mV of amplitude setpoint. (b) V_{cpd} profiles extracted from the corresponding V_{cpd} image (along the full length of the scanned area) for different DC sample biases, without illumination, and superimposed to the corresponding height profile. FM-KPFM parameters : frequency and amplitude of the electrical excitation : 958 Hz and 600 mV.

Such a measurement allows observing the V_{cpd} step across the ITO/HHJ step for different biases applied to the ITO electrode. The absence of floating potential is expected to lead to a conservation of the V_{cpd} step, with a global shift of the entire

V_{cpd} profile according to the DC sample bias value (up-shift for positive bias and vice versa). In order to visualize this V_{cpd} step profile for each applied bias, Figure S1b shows cross-sections extracted from the corresponding V_{cpd} image along the total length of the scanned area, at different lines corresponding to different DC sample biases, without illumination. A cross-section extracted from Figure S1a illustrates the typical height profile corresponding to the V_{cpd} cross-sections. The measured amplitude of the V_{cpd} step averaged over the 6 profiles is (405 ± 11) mV. The small standard deviation of the average value of the V_{cpd} step (inferior to 3%) indicates negligible variations of the V_{cpd} step with the magnitude of the applied bias on the ITO electrode. This shows the absence of a floating potential across the ITO/HHJ stacking.

Figure S2 shows the superimposed profiles of the FM-KPFM height and V_{cpd} recorded in UHV along a given scan line over a nano-structured TiO_2 film. The measurement was performed successively with and without illumination. It can be seen that negligible change in the topography appears, ensuring the absence of light-induced artefacts in the topography recording. A very good matching of the V_{cpd} profiles is observed as well: only small discrepancies not exceeding 25 mV are measured between the two curves. This value is negligible in relation to the overall variation amplitude of the V_{cpd} signal across the image. On a more general view, the averaged V_{cpd} values without illumination and upon illumination are (905 ± 58) mV and (890 ± 55) mV, respectively. The difference between the averages is contained within the standard deviation. We can therefore conclude that, if any photovoltaic effect intrinsic to the ITO/ TiO_2 system is present, it is very small.

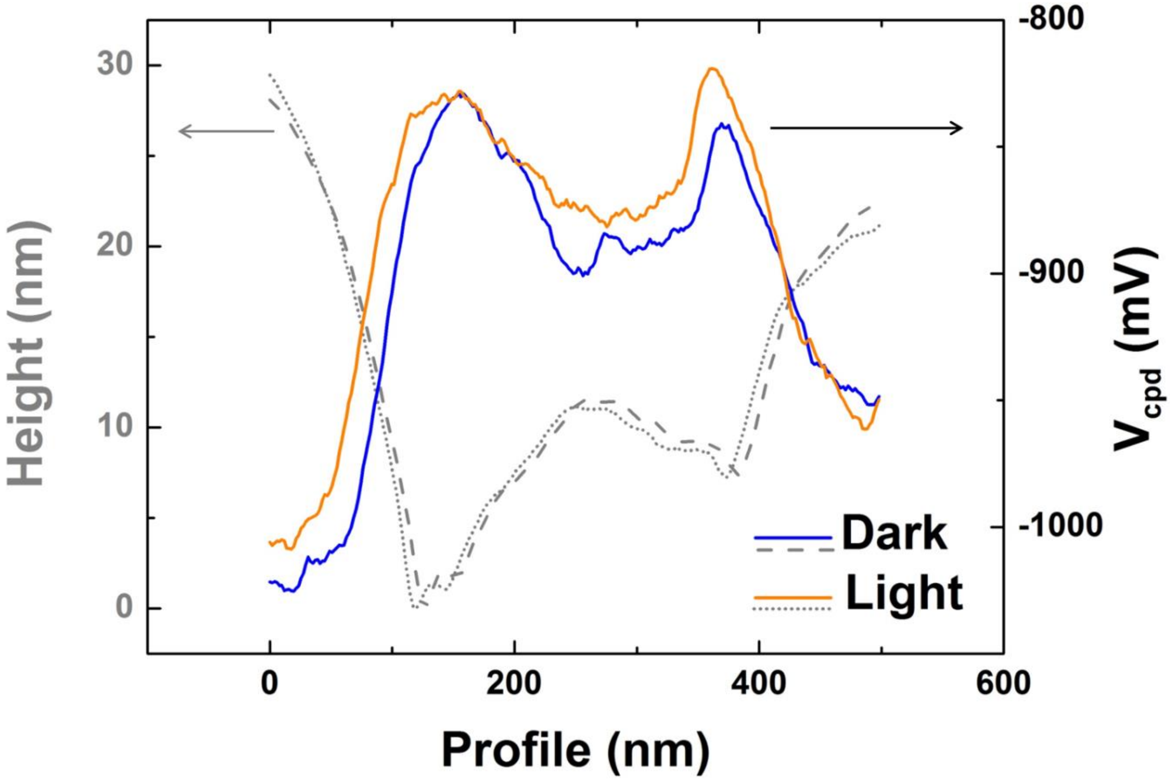

Figure S2: FM-KPFM height and V_{cpd} profiles over a nano-structured TiO_2 film deposited on an ITO electrode, recorded with and without illumination. The measurement was performed in UHV. FM-AFM parameters: -22 Hz of frequency shift setpoint and 40 mV of amplitude setpoint. FM-KPFM parameters: frequency and amplitude of the electrical excitation : 958 Hz and 600 mV.
